# Is Kummell’s Disease a Misdiagnosed and/or an Underreported Complication of Osteoporotic Vertebral Compression Fractures? A Pattern of the Condition and Available Treatment Modalities

**DOI:** 10.3390/jcm10122584

**Published:** 2021-06-11

**Authors:** Olga Adamska, Krzysztof Modzelewski, Artur Stolarczyk, Jurij Kseniuk

**Affiliations:** 1 Collegium Medicum, University of Zielona Góra, 28 Zyty St., 65-046 Zielona Góra, Poland; 2 Orthopaedic and Rehabilitation Department, Medical University of Warsaw, 61 Żwirki i Wigury St., 02-091 Warsaw, Poland; krzysztof.modzelewski@wum.edu.pl (K.M.); drstolarczyk@gmail.com (A.S.); 3 Carolina Medical Center, 78 Pory St., 02-757 Warsaw, Poland; xwz12@icloud.com

**Keywords:** Kummell’s disease, intravertebral vacuum cleft, vertebral body collapse, vertebral osteonecrosis, surgical treatment

## Abstract

This narrative review provides the outcomes of minimally invasive surgery (MIS) and describes the available conservative treatment options for patients with osteoporotic vertebral compression fractures (OVCFs) that have risk factors for Kummell’s disease (KD). It aims to explore the evidence, emphasize the possible therapy complications, and aims to propose the most efficient clinical strategies for maintaining a good overall condition of individuals who may suffer from neurological deficits from a late-diagnosed OVCF complication. The secondary objective is to sum up the diagnostic particularities concerning individuals prone to OVCFs and KD, as the major risk factor for developing these severe conditions remains osteoporosis. Findings of our narrative review are based on the results found in PubMed, Embase, and Google Scholar from the beginning of their inception to December 2020, described independently by two authors. All of the studies included in the review focus on reporting the following treatment methods: conservative methods, vertebroplasty, kyphoplasty, targeted percutaneous vertebroplasty, frontal and side-opening cannula vertebroplasty, SpineJack, bone-feeling mesh container treatment, and the difference in the cement viscosity used (high vs. low) and the approach used (unilateral vs. bilateral). The comparison of randomized control trials (RCTs) as well as prospective and retrospective case series showed a comparable efficacy of kyphoplasty and vertebroplasty, and described cement-augmented screw fixation and the SpineJack system as effective and safe. Although it should be noted that several studies revealed inconsistent results in regards to the efficacy of using back braces and analgesics in patients who had vertebral fractures that were overlooked or not enrolled in any active surveillance program to track the patient’s deterioration immediately. Nevertheless there are non-standardized guidelines for treating patients with OVCFs and their complications already established. Using these guidelines, a treatment plan can be planned that takes into consideration the patients’ comorbidities and susceptibilities. However, the primary approach remains the management of osteoporosis and that is why prophylaxis and prevention play a crucial role. These measures reduce the risk of disease progression. Unfortunately, in the majority of cases these measures are not taken into account and KD develops.

## 1. Introduction

Current literature states that the incidence of KD is around 7% to 37% in elderly individuals. The population in developed countries is aging and KD is a potential complication in up to one-third of OVCFs, which requires discussing an appropriate therapeutic approach for these patients [1,2,3,4,5,6,7,8,9,10,11,12,13,14]. KD usually affects the lower thoracic or upper lumbar region of the spine and it usually involves only a single vertebra [15]. The majority of injuries remain asymptomatic. Thus they are frequently undiagnosed since only 23 to 33% of all fractures are clinically evident [16].

In the female age range of 50 to 54 around 10% of the patients suffered from at least 1 OVCF, but after the age of 80 statistics revealed a sudden spike to 50%. This analysis emphasizes a big role of prophylactic measures, watchful waiting, and the observation in an aging population that is at risk of osteoporosis or one with an OVCF. Prophylaxis should be implemented to minimize the prevalence of altered healing, nonunion, intravertebral vacuum clefts (IVC), and the most emerging and dangerous complication of vertebral fractures that is KD. These complications reduce the patient’s quality of life (QoL) and therefore require adequate preventive measures.

## 2. Materials and Methods

While searching for relevant studies throughout the database, we used the following keywords: osteoporotic vertebral compression fractures; vertebral fractures; post-traumatic osteonecrosis; delayed vertebral collapse; avascular osteonecrosis and treatment. The inclusion criteria consists of:

(1) study design: randomized control trial, prospective/retrospective cohort studies
(2) population: patients suffering from osteoporosis—vertebral fractures diagnosed with an imaging study
(3) intervention: MIS or conservative treatment
(4) language: articles originally published in English.

Studies that have met our inclusion criteria are found in Table 1, Table 2 and Table 3. Table 1 shows all of the relevant information:

(1) authors
(2) year of publication
(3) journal
(4) study design
(5) study type
(6) group abundance
(7) the level of OVCFs
(8) technique of treatment

We extracted from the data and included in the Table 2 and Table 3:

(1) age
(2) gender
(3) bone mineral density (BMD)
(4) numbers of treated injured levels
(5) technique used as a treatment method
(6) properties of each chosen method
(7) benefits of each chosen method
(8) the results and the efficacy
(9) percentage of complications
(10) adjacent vertebral fracture
(11) kyphosis
(12) pain progression

## 3. Epidemiology

When a patient presents with a fracture, the fracture becomes an independent risk factor to the BMD in order to facilitate skeletal alterations. In a young population, a BMD below the population average increases the risk of fracture by around 60%. However elderly patients with multiple comorbidities and a decreased BMD are at an even greater risk [16]. Studies report that the risk of developing nonunion from OVCFs ranges from 13.5% to 19.6% [17,18]. Whenever fractures fail to fuse, the patient’s pain increases, the QoL decreases, and even neurological deficits can occur. Therefore it is crucial to have regular check-ups which will evaluate the risk for nonunion which will decrease the incidence of any further serious complications. Current literature provides us with the evidence that justifies the use of radiological studies as a predictor of delayed union [17,18,19]. Prospective cohort studies found that magnetic resonance imaging (MRI) can detect nonunion 6 months after an OVCF [19,20]. Inose H. et al. in their prospective cohort study from 2020 proposed the following radiological risk factors for nonunion 12 months after an OVCF:

(1) a middle column injury
(2) a diffuse low-intensity T1-weighted MRI pattern
(3) a fluid-intensity and diffuse low-intensity T2-weighted MRI pattern [21].

Additional risk factors are: the female sex, having a low body mass index (BMI), smoking, a sedentary lifestyle, low calcium intake, and frequent falls [16].

## 4. Pathophysiology

KD is an eponym for a delayed post-traumatic bone osteonecrosis. Patients usually present with advanced stage kyphosis in the thoracolumbar (T-L) area within months to years after experiencing a minor trauma, initially presenting without any symptoms [22]. Possible causes include avascular osteonecrosis, microfracture, atrophic nonunion, and a nutritional injury fracture. Kummell H. found that an OVCF does not lead to KD in every case. The nutritional bone status plays a big role in disease progression. Angiography of the spinal arteries has shown occlusions in some patients with IVC [23]. Hematopoietic cells become necrotic within 12 h after the onset of ischemia. Without reperfusion, the avascular necrosis develops into KD [23].

## 5. Risk Factors

Osteoporosis is the biggest risk factor for KD, hence its high frequency in the affected patient population. Osteoporosis is found in more than 20% of the population over 50 years of age and appears more often in slim white females [22].

The idiopathic origin of KD may be due to drug cytotoxicity, avascular necrosis, and decreased intraosseous blood flow [24].

Post-traumatic avascular necrosis is the most frequent factor causing decreased blood supply to the bone marrow. Any damage that can obstruct the vessel supplying the vertebral body can lead to non-healing and osteonecrosis.

There are a variety of conditions that can obstruct the artery lumen supporting the spine: sickle cell crisis, Gaucher’s disease, Caisson disease, SLE, prothrombotic states, pancreatitis, lipolytic enzymes, fluid overload, dyslipidemia, leukemia, lymphoma, diabetes mellitus, sarcoidosis, cirrhosis, hyperuricemia, malignancies, prolonged corticosteroid use (at least 5 mg per day over a period of 3 months), and alcohol abuse [25,26].

## 6. Clinical Presentation

Initially the description of KD included 3 stages. In stage 1 patients are exposed to trauma. In stage 2 patients develop back pain within months to years after the initial trauma. In stage 3 the patient develops kyphosis [27].

Following studies reported that the onset of the condition is symptomless regardless of the type of injury. Radiological changes were rarely detected. The second stage consists of back pain without any significant limitations, and afterwards patients develop a strong back pain localized in the area of injury. Ultimately persistent pain and kyphosis then appear, which may be associated with spinal cord compression causing even more severe neurological compromise. If symptoms do occur, patients complain of excruciating pain and neurologic symptoms such as tenderness. With further disease progression, thoracic kyphosis develops and the patient’s height decreases [28].

Another clinical evidence is IVC, which is the characteristic for KD, described by Maldague et al. for the first time. It appears as a transversal radiolucent line-like shadow in the region of the collapsed or adjacent vertebrae in a CT scan [26]. Studies revealed that the incidence of nonunion in patients suffering from KD is approximately 13.5% and the incidence of IVC is about 7–13% [29].

### Consequences of Delayed Vertebral Compression Fractures

There are several concerns associated with KD: impaired gait & balance, decreased QoL, loss of independence, depression, mental breakdown, and an increased mortality rate [30]. The collapsed vertebral body and progressive kyphosis lead to a reduced volume in the thoracic and abdominal cavities. This further deteriorates lung function—even in patients with asymptomatic OVCFs and nonsmokers.

Studies confirmed a higher mortality in patients with present OVCFs compared to those who had suffered from low BMD in the postmenopausal age without any existing vertebral abnormalities [31].

The treatment of KD is controversial. The problem of whether to operate or not remains. The European Vertebral Osteoporosis Study enrolled people with vertebral compression fractures [32]. The results revealed that the first fracture resulted in a 4-fold increased risk of fracture in the adjacent vertebrae and a 2 to 3-fold increased risk of fracture in a different location [33]. In patients in whom neurogenic pain occurred together with a mild kyphosis, MIS had the highest success rate. The use of conservative methods can delay the progression, but they do not decrease the risk of KD. The aim of the treatment is to diminish the progression of the disease. At the same time it should stop the gradual deterioration and minimize the risk for its development by using operative solutions [34]. The World Health Organization (WHO) has a 10-year fracture risk assessment calculator (FRAX) for different population groups. Based on the FRAX score, OVCF therapy is decided upon [35]. According to a systematic review published in 2017 describing the risk factors for the failure of conservative treatment in 1203 patients with OVCF, a fracture in the T-L region increases the risk of nonunion [36]. This study also revealed that even though conservative management leads to good outcomes in the majority of cases, patients that had impaired healing and those who had fractures in the T-L region are likely not to benefit from nonsurgical methods. These cases are strongly related to a poor recovery prognosis, prolonged back pain, decreased activities of daily living (ADLs), and a higher risk for the fractures in adjacent vertebrae [36].

## 7. Diagnostic Methods

Radiological evaluation is recommended for all patients in whom there is a suspicion of spinal trauma. CT scans provide detailed information about bone injuries and are mainly used to establish a final diagnosis and to classify the fracture [34,37]. In the final step, an MRI is performed to assess the trauma. It provides additional information about the soft tissue damage. This tool is helpful in estimating the severity of the existing OVCF and seems to be especially beneficial in investigating asymptomatic injuries [37]. The severity of patient’s condition is assessed based on one of the following scales: AO Surgery Reference [38] or the Spine Trauma Group scale, known as the thoracolumbar injury classification and severity score (TLICS), sometimes described as the thoracolumbar injury severity score (TISS) [39].

## 8. Treatment

Because the majority of OVCFs are type A or type B, the aforementioned scales primarily point towards conservative treatment, with the greatest focus being on the maintenance of the patient’s health when the disease is not significantly advanced [30]. Osteoporosis is a serious disease with devastating complications [40]. KD is one of those complications facilitating failure of the fracture healing process and therefore the decision on the invasiveness of the therapy must be appropriate for the injury. In the case of patients with KD with persistent pain and no neurological symptoms, Li J.B. et al. in a study from 2020 recommend MIS as a first choice [41]. When an ischemic area develops, it eliminates any healing potential and facilitates nonunion [42]. Jang J.S. et al. and Stallenberg B. et al. established that patients with OVCFs with nonunion and back pain with or without symptoms of neurogenic compression are candidates for surgical stabilization [43,44]. Studies consistently show that up to a third of the patients will unfortunately not respond successfully to conservative therapy alone when dealing with OVCFs [45]. Since the efficacy of MIS has been proven for individuals suffering from KD, there is no reason to prolong the time to treatment. Even though patients with KD are elderly and suffer from multiple comorbidities, MIS reduces iatrogenic tissue trauma due to smaller incisions, decreases soft tissue damage and blood loss, and reduces muscular and ligamentous rupture. [46].

### 8.1. Conservative Treatment

**Figure 1:**
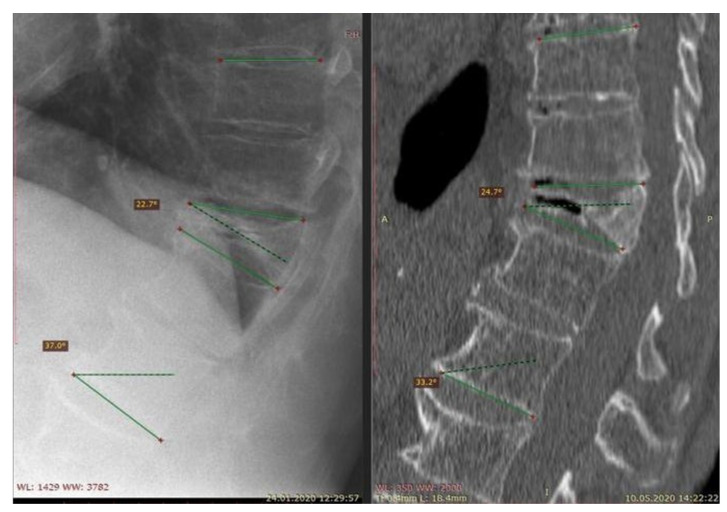
Images (from the left a X-ray and a CT scan respectively) of a vertebra injured from OVCF after conservative treatment failure.

Over-the-counter pain medications are often effective in pain management, but they do not facilitate the healing process. A treatment needs to be introduced to reduce the risk of subsequent fractures. Therefore, bone-strengthening drugs such as bisphosphonates and hormone replacement therapy may be prescribed to stabilize and restore the bone density [47]. Reducing the range of motion (ROM) with a back brace helps in weight-bearing and decreases postural flexion. Nevertheless as shown in Figure 1., it is controversial whether bracing is effective in the treatment of spinal injury and if it provides better outcomes [48].

#### Complications of Conservative Treatment

The usual adverse effects of pharmaceuticals used by patients with KD that affect the central nervous system can lead to cognitive impairment, sedation, and constipation, especially in elderly patients. NSAIDs and acetaminophen use causes gastritis, gastric ulcers, hepatic and renal problems; especially in large doses, which can eventually lead to the worsening of the overall condition of the patient. Bed rest may result in an even greater BMD and muscle strength loss. The lack of physical activity can also lead to an impaired cardiovascular system and pulmonary function results [49]. Conservative treatment has been discussed for a long time and the controversy of its usage has yet to be solved. However most literature agrees on its rather low value in improving the patient’s overall condition. There are not that many studies that clearly mention the complications of conservative therapy, but many physicians concur that patients using back brace are prone to back muscle atrophy, as stated by Mazanes D. et al. in a systematic review from 2003 and Kondo L. et al. and Dang S. [30,50,51].

### 8.2. Surgical Treatment 

The decision to perform surgery in order to improve the patient’s condition and to prevent further deterioration of the patient’s health is made in the following events: conservative management is ineffective; there are neurological complications; there is pain limiting the patient’s ROM; and when there is a significant degree of kyphosis and neurogenic claudication [32]. A surgical approach for spinal fractures is still not widely accepted by orthopedic surgeons, however there are no established guidelines. Nevertheless, studies show that surgical treatment leads to a large improvement in the condition of the patients who are prone to adverse events. In a majority of the cases, surgery is chosen [51]. The European Spine Society, the European Spinal Deformity Society, and the European Section of the Cervical Spine Research Society reported the world-wide prevalence of osteoporosis and its complications. It concluded that a high risk of increased morbidity and mortality exists. It suggests surgery to be used as a modern treatment option, this recommendation is based on evidence based medicine (EBM). After the development of a severe neurological compromise due to kyphosis, ranging from paraplegia to paraparesis, the chance of recovery to a satisfying level is low [41]. MIS is less traumatic than conventional open surgery, potentially resulting in a faster recovery while providing similar clinical mid to long-term results. Percutaneous vertebral augmentation (PVP and BKP) has an important role in achieving pain relief and improving clinical outcomes and has been well argued in most of the cases [52,53,54]. Bone cement-augmented pedicle screw fixation and the SJ system are other options for restoring spinal stability, alleviating debilitating pain, and improving clinical outcomes [55].

## 9. Results

A summarized comparison of the patient’s demographic, clinical, and radiological data, as well as the therapeutic methods used are presented in Table 2 and Table 3.

The RCT conducted by Hansen E. et al. investigated whether PVP achieves better results than the placebo [1]. The outcomes show a statistically significant improvement in back pain, primarily in forward bending in patients undergoing the surgery. Zhu Y. et al. compared BKP to PVP [2]. Both of the augmentation methods resulted in a satisfying improvement. BKP however had lower rates of cement leakage.

Lou S. et al. compared pain scores in patients who were treated with PVP and the placebo. Over time a tendency toward an increasing effect of PVP was maintained. For the open-label studies, PVP significantly reduced pain. New vertebral fracture risk was similar in both groups [3].

Furthermore, targeted PVP appeared to achieve lower skin positioning fluoroscopy times and lower total fluoroscopy times. It used a lower dose, had a shorter operation time, and was more precise than traditional PVP [4]. Targeted PVP also revealed a lower incidence of cement leakage. Figueiredo N. et al. compared FOC and SOC and visualized better outcomes for VAS for SOC in the follow-up period, with similar pain severity at onset. Cement leakage was reported frequently after FOC [5].

A study analyzing the efficacy of unilateral and bilateral PVP reported a significant improvement of VAS and ODI scores without any significant differences in between both of them, although bilateral PVP caused a significantly higher percentage of cement leakage [6].

A BKP vs. SJ comparison performed by Noriega D. et al., revealed overall similar efficacy of both procedures. Vertebral body height restoration and kyphosis correction was better with the SJ procedure in a 3-year follow-up [7]. PVP was studied with low viscosity bone cement and high viscosity bone cement. There was a marked improvement in the VAS, ODI, kyphosis, Cobb’s angle, and vertebral height noted in both the groups, and there were no significant differences between the two groups. Cement leakage was seen less with the usage of high viscosity bone cement [8]. Schwarz F. et al. questioned if the newer generation vertebral access devices for BKP provided better performance. They were meant to reduce the length of the operation, however this hypothesis was not proven. Furthermore the results showed a prolonged irradiation duration in comparison to traditional vertebral access devices [9]. BKP with the conservative treatment achieved significantly better outcomes. The predominance of BKP was especially significant in a better QoL, normalization in kyphotic angulation, and pain alleviation, although there was a greater risk of adverse effects to this surgical approach. Van Meirhaeghe J. et al. concluded that the risk can be reduced with more accurate positioning of the patient during the procedure [10]. According to Yang S. et al., PVP and BKP did not differ significantly in the VAS, vertebral height, kyphotic angle, and QoL. Complications after surgery appeared in both groups and mainly included bone cement leakage and adjacent vertebral fractures [11].

Unilateral and bilateral PKP brought promising effects for the improvement in vertebral height, Cobb’s angle, VAS, operation time and lower cement injection volume. Unilateral PKP is characterized by shorter operation time, lower hospital costs, lower exposure to radiation and less bone cement volume. On the other hand, bilateral PKP shows a lower risk of the adjacent vertebral body fracture compared with the unilateral PKP. Here the benefit-risk ratio is inconsistent [12]. A RCT comparing specifically BFMC and KP resulted in significant pain relief and kyphosis correction in favor of BFMC [13].

## 10. Discussion

PVP is considered a treatment option for OVCFs and KD. It is an image-guided procedure using a bone cement injection made from polymethyl methacrylate (PMMA) [54,55]. However it has a few postoperative risks: cement leakage into the spinal canal, dislocation of bone fragments, and posterior wall displacement [56].

The most likely side effect, cement leakage, can be eliminated by BKP with a high success rate. It involves the inflation of a balloon catheter inside the collapsed vertebral body which causes the restoration of the vertebral height with a more viscous bone cement when compared to PVP. This allows for a lower pressure of injection, thus considerably reducing the risk of leakage. Both methods are safe and effective for the treatment of vertebral body compression, but neither are perfect. The PMMA hardens quickly and behaves as a cast [57,58], especially in patients with an IVC.

Studies are inconsistent when looking at their efficacy [59], however callus formation in the patients with KD treated with the percutaneous vertebral augmentation appears as well as a greater rate of osteolysis. This may lead to the displacement of the bone cement even in KD without neurologic deficits [60,61].

The PMMA, broadly used in orthopedic surgery, remains one of the most convenient materials used [62]. However its performance in a high compression environment and weak bonding to bone makes it somewhat controversial. A mineralized collagen (MC) to improve the physical properties of the cement when incorporated into the PMMA (MC-PMMA) is being tested [63]. It consists of a better bioactive composite and the ability to augment an implant in an intervertebral cavity. It decreases the pressure given to the joint, which facilitates pain and fatigue relief. A better postoperative effect is seen because of bone reinforcement. The Up-To-Date Overview states its advantageous effect on osteoporotic bones [64], and the fact that it significantly diminishes stress during screw-augmentation [65]. Its role is to achieve greater stability and alignment of the vertebral column, thus being beneficial for the patient, delaying vertebral body collapse and progress to nonunion. Although it brings the risk of neural injury due to leakage into the spinal canal, pulmonary embolism caused by cement migration into the external venous plexus, and irremovable hardware, it is believed these complications can be avoided with good surgical technique and with the good accuracy of the injection [66].

Bone cementing is accompanied by percutaneous short-segment pedicle screw fixation. This procedure is indicated in patients with KD complicated by myelopathy, compression of the spinal cord, destabilization, and those without any neurological deficits [67]. Stability of the screws is a priority, because their loosening is relatively frequently seen and this causes high rates of infection and may result in instrumentation failure [68,69]. Cement-augmentation has been utilized to stabilize osteoporotic bone fractures [70]. It allows for the fixation of screws in a fractured bone and even reduces the risk of a possible infection due to its antibiotic component. Studies conducted by Park J.S. et al., Cho Y. and Huang Y.S. et al. confirm the efficacy of this method in patients with KD [67,71,72]. Tang Yc. et al. analyzed percutaneous cement-augmented pedicle screw fixation in osteoporotic spine with lumbar degenerative disease and concluded that it is a beneficial option for elderly individuals (average age 78) because of its advantageous properties in weakened bones. Due to the biomechanical stability, pedicle screw fixation can be performed in osteoporotic spines and weakened bones. In an effort to rule out the most undesirable side effects for this method, these preventative methods should be introduced: (1) use of high viscosity cement; (2) reduced volume of cement; (3) setting a trajectory and the size of pedicle screws using a preoperative CT scan; (4) cement injection with small doses and a slow speed (5) fluoroscopic control of the injected cement. The infection rate (6,52%) was seen in patients with diabetes and they did not need revision surgery [73]. However, in the case of an untreatable surgery site infection, component extraction is urgently needed. This corrective surgery is unwanted because the extraction torque used on the fixed screws in on osteoporotic bone may facilitate further damage to the already fractured vertebral body. Nevertheless, extraction techniques of fenestrated screws with small diameter fenestration holes are considered to carry a low risk. Goetzen et al. confirmed that uncomplicated revision surgery can be achieved without the need for any special instrumentation or enhanced torque for fenestrated screw removal. Additional damage was not seen at the bone-cement interface [74].

A study investigating tissue preservation did not reveal an increase of osteoporotic bone degeneration after the removal of cement-augmented fenestrated screws. The cement screw head was fragile enough to break off during the removal of a component [75]. However, Bullmann et al. mentioned that non-augmented screws allowed for a significantly higher axial pull-out strength and torque. They also noted that revision surgery increases an objective chance of cement leakage if cement augmentation is reintroduced [76]. Unfortunately there are many limitations associated with the risk assessment of revision surgery. Most studies are made using a cadaver. As such, the clear outcomes clarifying the issues of osseointegration and bone remodeling are not applicable due to the use of cadaveric bones that lack any healing potential [77].

Huang Y.S. et al. suggest that percutaneous cement-augmented pedicle screw fixation as an effective treatment method for patients suffering from KD with multiple comorbidities and/or severe osteoporosis [72]. A high success rate was found eliminating any intervertebral instability; which is a significant factor of delayed neurological deficits following vertebral body collapse. Further advantages are: (1) screw stress and loosening risk is minor, (2) shorter operation time, (3) less blood loss, (4) stabilization with an extremely low misplacement rate and low morbidity [70,71,72,73,78], (5) MIS for elderly patients with comorbidities, who are not able to withstand open spine surgery [46,79,80,81].

In a prospective multi-center clinical trial, Noriega D. et al. introduced the results using a SJ implant for the treatment of OVCFs [55]. The x-ray guidance allows a thin hollow needle to be placed in the spine and allows the injured vertebral body to be filled. The expansion of the implant allows for the restoration of the prefracture height [82]. Satisfactory height restoration and deformity correction may reduce the incidence of possible future spinal fractures which are relatively frequently seen in patients with a history of OVCFs and provide better clinical outcomes and an improvement in the QoL of the patient [82,83]. Furthermore, back pain is reduced in the 12-month follow-up period. The reduced need of analgesics within 48 h after the surgery is another benefit of the SJ. The radiological outcome reveals a significant improvement of the kyphotic angle within 48 h of surgery. No implant-related complications were reported and no component removal was performed. A statistically insignificant amount of patients (2.9%) experienced procedure-related complications. Adjacent fracture events 1 year after surgery were reported at only 2.9%. The SJ appears to be an effective low-risk procedure for patients with traumatic vertebral compression fracture allowing for a fast and substantial improvement in the patients’ QoL [55,83]. These results bring an optimistic prognosis for OVCF and KD treatment. However, there is currently no evidence showing that the outcomes achieved can be seen in patients with KD, likewise there is no evidence in regards to any adverse outcomes.

**Figure 2:**
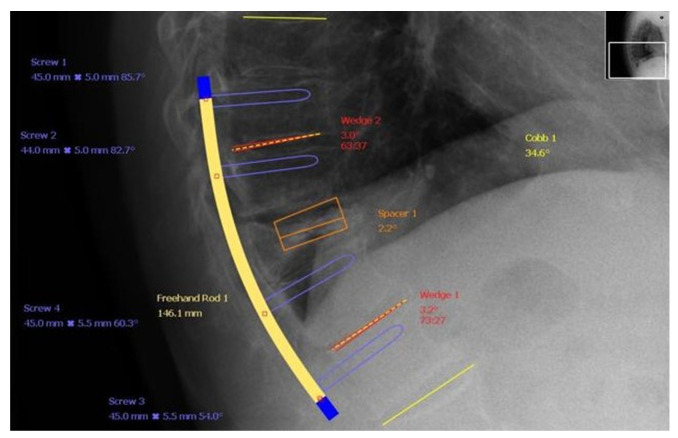
A preoperative visualisation of the effects of MIS treatment for KD developed from OVCF after an ineffective use of conservatice regimen.

**Figure 3:**
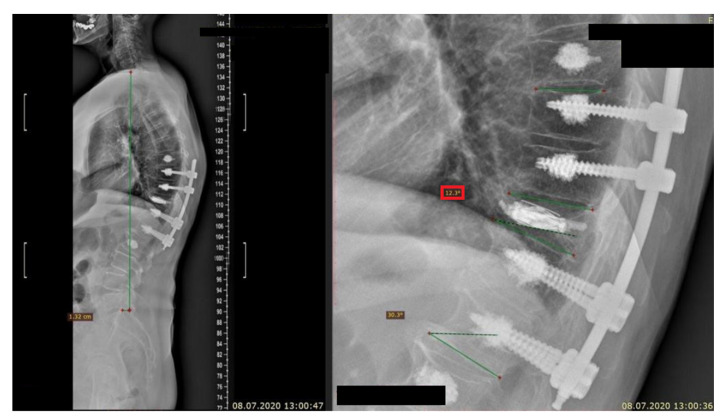
Imagining of a vertebral body height restoration achieved with an introduced surgical approach, consisting of SJ system, accomplished by PVP and minimally invasive percutaneous transpedicular fixation using bone-cement screw augmentation.

No standard or preferred treatment for KD exists. Delayed vertebral body collapse needs to be considered in any patient with recurrent or worsening back pain. For KD stages I and II, kyphoplasty and vertebroplasty can be used to achieve good pain relief, vertebral body height restoration, and kyphosis deformity correction immediately after the surgery and with a decrease at a follow-up visit. For stage III patients that have spinal canal stenosis (especially those with nerve damage), percutaneous vertebral augmentation treatments are not effective, having a great risk of cement leakage with the potential risk of severe neurological damage. Therefore a safe, effective, and less invasive treatment approach for KD is needed. Satisfactory results have been achieved with bone cement-augmented percutaneous short-segment screw fixation in patients with severe osteoporosis in retrospective reviews [67,71,72,84,85,86,87,88,89,90], which also confirm the imaging from Figure 2 and Figure 3.

In the paper conducted by us previously, there is a case of a patient who developed KD when using bracing devices for over 2 years and was eventually qualified for a surgery using multiple from a variety of MIS procedures [91]. This emphasizes that the assessment of benefits and harms of any treatment approach should be gradually maintained to allow an interference in a timely manner. What is more, the aforementioned Figure 2 and Figure 3 evidence that the theoretical assumptions agreed with the actual patient’s condition after a meticulously planned surgical approach. 

## 11. Conclusions

There is limited available evidence and data in reference to the treatment methods considered for patients with OVCFs who are prone to complications. The prophylaxis for patients with risk factors for spinal problems and decreased BMD should be a priority, but at the moment no preferred treatment patterns for KD exist. CT images of the spine in the elderly should be evaluated with great accuracy and frequency to yield improvement in prevention of future fractures and neurological complications. Otherwise it positively correlates with QoL and ADLs.

KD should be suspected in any patient with recurrent or worsening spinal symptoms who present with some clinical characteristics. Early detection of spinal alteration enhances the chance for a successful therapy and diminishes the risk of further complications.

Largely, conservative treatment regimens have usually been classified as less effective than surgical approaches. They are proven to facilitate delayed neurological deficits, but since patients with osteoporosis suffer from KD simultaneously, we should pay marked attention to the best possible therapy because of the decreased healing potential in patients suffering from KD.

When pain is the only complaint, the objective is to eliminate it at the fracture site and restore stability of vertebra. For stages 1 and 2 of KD, BKP and PVP have achieved good pain relief, vertebral body height restoration, and kyphosis deformity correction immediately.

For stage 3 patients presenting with spinal canal stenosis, especially those with nerve damage, percutaneous vertebral augmentation methods are not effective, bringing the risk of cement leakage, with the potential risk of severe neurological damage. However literature suggests that cement-augmented screw fixation combined with the SJ system shows promise as a safe method for treating KD. A satisfactory correction of spinal kyphosis and vertebral height with pain relief and an improvement in neurological functions with the stability of the vertebral column can be achieved. Thanks to the additional intravertebral components, there is a low risk of cement displacement.

## Figures and Tables

**Table 1:** Characteristics of eligible studies.

[Reference Number] Authors	Date of Publication	Journal	Study Design	Clinical/Non-Clinical	Group Abundance	Level of Fracture Appearance	Technique of Treatment	Source	Accessed Date
[1] Hansen E.J.	September 2019	Integrative Journal of Orthopaedics and Traumatology	RCT	clinical	46	T5-L5	vertebroplasty vs. placebo	https://www.researchgate.net/publication/336149174_Vertebroplasty_vs_SHAM_for_Treating_Osteoporotic_Vertebral_Compression_Fractures_A_Double_Blind_RCT	21 January 2021
[2] Zhu Y. et al.	November 2019	Medicine	RCT	clinical	1077	NR	vertebroplasty vs. kyphoplasty	https://journals.lww.com/md-journal/FullText/2019/11080/Therapeutic_effect_of_kyphoplasty_and_balloon.28.aspx	21 January 2021
[3] Lou S. et al.	December 2019	Osteoporosis international	RCT	clinical	1624	T5-L5	vertebroplasty vs. conservative treatment/SHAM	https://pubmed.ncbi.nlm.nih.gov/31375875/	21 January 2021
[4] Xu J. et al.	October 2019	Surgical innovation	RCT	clinical	42	T10-L4	targeted percutaneous vertebroplasty vs. traditional percutaneous vertebroplasty	https://pubmed.ncbi.nlm.nih.gov/31167616/	21 January 2021
[5] Figueiredo N. et al.	June 2009	Arquivos de neuro-psiquiatria	RCT	clinical	47	T4-L5	frontal vs. side- opening cannula vertebroplasty	https://pubmed.ncbi.nlm.nih.gov/19623429/	21 January 2021
[6] Chen C. et al.	December 2014	Journal of spinal disorders and techniques	RCT	clinical	39	NR	unilateral vs. bilateral vertebroplasty	https://pubmed.ncbi.nlm.nih.gov/24901876/	21 January 2021
[7] Noriega DC. et al.	March 2019	Osteoporosis International	RCT	clinical	30	T7-L3	kyphoplasty vs. SpineJack	https://pubmed.ncbi.nlm.nih.gov/30488273/	21 January 2021
[8] Zhang L. et al.	February 2015	Clinical neurology and neurosurgery	RCT	clinical	32	NR	high viscosity vs. low viscosity cement vertebroplasty	https://pubmed.ncbi.nlm.nih.gov/25524481/	21 January 2021
[9] Schwarz F. et al.	November 2019	Archives of orthopaedic and trauma surgery	RCT	clinical	65	L1-L4	early versus newer generation vertebral devices access for kyphoplasty	https://pubmed.ncbi.nlm.nih.gov/31278508/	21 January 2021
[10] Van Meirhaeghe J. et al.	May 2013	Spine	RCT	clinical	300	NR	kyphoplasty vs. nonsurgical methods	https://www.ncbi.nlm.nih.gov/pmc/articles/PMC3678891/	21 January 2021
[11] Yang S. et al.	July 2017	Acta orthopaedica et traumatologica turcica	SR based on RCTs	clinical	850	NR	unilateral vs. bilateral vertebroplasty/kyphoplasty	https://pubmed.ncbi.nlm.nih.gov/28647158/	21 January 2021
[12] Tang J. et al.	October 2019	Journal of the College of Physicians and Surgeons	SR based on RCTs	clinical	178	T11-L2	unilateral vs. bilateral balloon kyphoplasty	https://pubmed.ncbi.nlm.nih.gov/31564267/	21 January 2021
[13] Duan Z. K. et al.	November 2019	Archives of osteoporosis	RCT	clinical	40	T11-L3	Bone-filling mesh container vs. kyphoplasty	https://pubmed.ncbi.nlm.nih.gov/31741066/	21 January 2021

**Table 2:** Demographics and preoperative characteristics.

Reference	Mean Age (year)	Gender (M/F)	BMD T-Score	No. Levels Treated	VAS at Baseline	VAS at the Follow Up	Weighted Mean Difference (95% Confidence Interval)
[1] SHAM/ PVP	69 71	2/22 4/18	−2.2 −2.7	28/27	5.30 4.06	1.6 1.6	NR
[2] BKP/ PVP	70 72	117/419 119/422	NR	NR	NR	NR	−0.19 (−0.39, 0.01)
[3] PVP/ CG	73 82	602/212 680/130	NR	NR	7.5 8.8	NR	NR
[4] targeted PVP/ PVP	68.5	3/18 2/19	NR	NR	7.38 2.48	NR	NR
[5] SOC/ FOC	NR	NR	NR	22/25	8.04 7.92	1.05 1.36	NR
[6] UPVP/ BPVP	69.5 69	NR	−3.18/ −3.32	23/21	7.99 7.66	2.82 2.61	NR
[7] BKP/ SJ	68 68	13/2 11/4	NR	17/16	8.43 8.05	2.5 1.44	NR
[8] HV PVP/ LV PVP	75.5/75.8	2/12 3/15	NR	17/22	8.4 8.6	2.2 1.9	NR
[9] PKP VAD/RI/ST	67/74/74	9/21//15/17//10/19	−3.98/−3.54/−3.70	30/32/29	NR	NR	NR
[10] PKP/ CG	72.2 74.1	34/115 34/117	Normal: 28/20 Osteopenic:54/57 Osteoporosis: 53/51	188 151	6.79 6.93	2.7 4.35	NR
[11] UVP/BVP UKP/BKP	67.9	308/416 + 126 not differentiated	NR	ca 906	NR/3.11 NR/3.17	NR/2.16 NR/1.28	NR
[12] UBKP/ BBKP	72.3 73.9	26/57 32/63	NR	83 95	7.9 7.8	2.7 2.6	NR
[13]BFMC/ BKP	<60 <60	9/11 8/12	<−3.0	20 20	7.5 7	1.5 1	NR

Abbreviations: VAS—visual analogue scale; PVP—percutaneous vertebroplasty; BKP—balloon kyphoplasty; CG—control group (SHAM or conservative treatment); CM—conservative management; SOC—side-opening cannula; FOC—front-opening cannula; UPVP—unilateral percutaneous vertebroplasty; BPVP—bilateral percutaneous vertebroplasty; SJ—SpineJack; HV PVP—High viscosity percutaneous vertebroplasty; LV PVP—Low viscosity percutaneous vertebroplasty; VAD—vertebra access device; RI—the Joline RapidIntro Vertebra Introducer Device; ST—Joline SpeedTrack Vertebra Introducer Device.

**Table 3:** Efficacy of treatment methods analyzed in the studies included in the review.

Reference	Duration of Follow Up Period	Oswestry Disability Index	Height in the Middle of Injured Vertebrae	Cobb Angle	Mean Operation Time (min)	Re-Fracture of Adjacent Vertebral Bodies	Mean Cement Volume (mL)	Cement Leakage
[3] PVP/ CG	<36 months	NR	NR	NR	NR	16.43% 5.83%	NR	NR
[4] Targeted PVP/ PVP	NR	73.11/34.71 79.73/48.28	NR	NR	20.05 25.43	NR	4 ml	4.76% 42.9%
[5] SOC/ FOC	6 months	NR	NR	NR	NR	NR	5.5 6.3	27% 68%
[6] UPVP/ BPVP	NR	42.82/18.43 39.42/22.37	NR	NR	31.12 52.34	NR	3.17 4.36	45% 78.9%
[7] SJ/ BKP	36 months	65.4 59.9	86%/81% 82%/79%	−3.2° ± 4.3°/ −2.5° ± 4.4°	23 32	6.67% 6.67%	4.9 5.1	6.67% 0
[8] HV PVP/ LL PVP	24.5 months	73.9/29.8 75.5/32.8	29.7%/45.6% 32.8%/50.7%	20.8/14.8 19.3/14.9	41.8 44.8	29.4% 68.2%	3.4 3.5	35.7% 83%
[9] PKP VAD/RI/ST	NR	NR	NR	NR	31/28/29	NR	5.5/6/6	NR
[10] BKP/ CG	24 months	NR	8.2%/6% 0%/−2%	3.4°/3.1° 0°/0.8°	65	7.38% 4.63%	4.8	NR
[11] UVP/BVP UKP/BKP	<54 months	NR	−0.10, 95% CI, −0.42 to 0.23; SMD = 0.10, 95% CI, −0.35 to 0.55 SMD = −0.13, 95% CI, −0.43 to 0.17	SMD = −0.05, 95% CI, −0.28 to 0.18	NR	NR	NR	NR
[12] UBKP/BBKP	6 months	87.3/86.4 23.5/22.9	15.3/15.7 23.6/24.3	34.3/33.8 23.4/22.6	29.8 31.5	6.02% 7.37%	3.1 3.5	NR
[13] BFMC/ KP	6 months	75.45/11.75 75.5/12.75	NR	23.16°/16.79°	43.8 43.3	20% 25%	NR	5% 40%

## Data Availability

The data presented in this study are available in Table 1. The access to them is provided via the URL addresses.
